# A Cellular Automata Approach for the Modeling of a Polyamide and Carbon Aerogel Structure and Its Properties

**DOI:** 10.3390/gels6040035

**Published:** 2020-10-18

**Authors:** Natalia Menshutina, Igor Lebedev, Evgeniy Lebedev, Patrina Paraskevopoulou, Despoina Chriti, Igor Mitrofanov

**Affiliations:** 1 International Science and Education Center for Transfer of Biopharmaceutical Technologies, Mendeleev University of Chemical Technology of Russia, Moscow 125047, Russia; chemcom@muctr.ru (N.M.); e.a.lebedev@gmail.com (E.L.); ivmitrofanov@gmail.com (I.M.); 2 Inorganic Chemistry Laboratory, Department of Chemistry, National and Kapodistrian University of Athens, 15771 Athens, Greece; paraskevopoulou@chem.uoa.gr (P.P.); chritides@chem.uoa.gr (D.C.)

**Keywords:** modeling, aerogels, diffusion-limited cluster aggregation, cellular automata, Young’s modulus, polyamide aerogels, carbon aerogels, pyrolysis

## Abstract

In this work, a cellular automata (CA) approach was used to generate 3D structures of polyamide and carbon aerogels. Experimental results are used as initial data for materials’ digital representations and to verify the developed CA models. Based on the generated digital structures, a computer study of aerogels’ mechanical properties was conducted. The offered CA models can be applied for the development of new nanoporous materials such as aerogels of different nature and allow for a reduction in the amount of required full-scale experiments, consequently decreasing development time and costs of new material formulations.

## 1. Introduction

Development of new functional materials with certain properties is an important scientific direction, accelerated by growing requirements of different practical fields. Increasing markets and fast data exchange claim a creation of new tools, making R&D process faster and more efficient. Taking into account these modern tendencies, we tried to build new models focused on material design and present in this paper the results obtained within a case study of new perspective nanomaterials—polyamide and carbon aerogels.

Aerogels are a class of potentially valuable materials with high porosity and low density. Aerogel density can be as low as 0.003 kg/m^3^, and its porosity can exceed 90%. The specific surface area of aerogels varies from 100 to 2000 m^2^/g [1]. The amount of possible applications of aerogels, both scientific and industrial, is permanently increasing, as well as the volume of the aerogel global market, demonstrating promising growth over last 5 years. Aerogels are used as thermal and acoustic insulation materials, as substrates for catalysts, filtration materials, and carriers of active substances in the pharmaceutical industry [2,3].

Formulation of new aerogels is associated with a large number of different experiments, which make the development process very expensive and time-consuming. Utilization of computer models, which allow for the prediction of aerogel properties, can reduce required resources and time by carrying out numerical experiments instead of real ones.

Modeling of nanomaterial structures such as aerogels is a challenging task. Structure simulation can be performed in different ways considering two scales—the nanoscale or mesoscale. Both simulation types include various methods with certain pros and cons. Aerogel structure modeling can be based on the reproduction of a gelation process with certain assumptions, as well as on methods generating a material internal structure without considering physics of the production process [3].

The most common nanoscale structure modeling method is molecular dynamics (MD). This approach does not consider degrees of freedom and replaces them with interactions between atoms and molecules. MD allows for the prediction of evolution of interacting particles (atoms, molecules, granules, etc.) over time and for the determination of the corresponding physical properties of a system [4,5].

The main advantage of MD is that it provides a complete description of atomic system behavior for various conditions without the need to make assumptions regarding mechanisms and processes that determine the behavior and properties of a material. Molecular modeling is widely used to understand equilibrium or nonequilibrium properties of many physical systems [4,6,7,8,9,10].

Another method which is used for model systems at the nanoscale is ab initio calculation. This allows one to obtain accurate calculations for the studied system, but it requires a lot of computing resources. In [11], metal organic chemical vapor deposition of AlN on graphene was studied, and in [12], Intercalation of P atoms in fullerene-like phosphorus carbide was studied. Furthermore, molecular dynamics may be advanced with first principles methodology ab initio molecular dynamics (AIMD). AIMD can describe evolution of the solid structure system. This method provides good accuracy for system modelling and relatively low computational costs [13]. However, despite the fact that at present it is possible to model systems containing more than several thousand atoms [14], in most cases, dimensions of the simulated structures do not exceed hundreds of nanometers. It is often insufficient for practical applications.

Aerogels are nanoporous, mostly mesoporous materials. This means that it is necessary to consider larger scales to evaluate aerogel properties than can be modeled using MD. The method which can be applied to mesoscale structure simulations must allow modeling systems with linear dimensions of more than 1 μm.

The mesoscale can be defined as an intermediate level at which new phenomena arise with their own characteristic time scales [15]. The standard strategy for the transition from the nanoscale to the mesoscale method is to group atoms into larger objects called combined atoms or superatoms [16,17]. Aerogel structures, studied in the current work, at the mesoscale consist of spherical aggregated globules with different diameters. Therefore, the virtual structure of these aerogels can be obtained by modeling globule motion and their aggregation. Moreover, there is a number of different methods which allow for modeling the required behavior of globules. They can be divided in two groups: particle–cluster aggregation (PCA) and cluster–cluster aggregation (CCA).

In PCA methods, at the first step, clusterization centers are placed in the modelling field. Then, a single particle (for instance, a solid globule) is generated in a random place within the field and starts moving until it collides with a clusterization center when aggregation occurs. These steps (particle generation and movement) are repeated iteratively until the whole structure reaches the required porosity. The most common representative of the PCA family is diffusion-limited aggregation (DLA), a feature that represents a change in the particle’s movement direction at each step [18]. Tracking of a single particle within one cycle makes PCA methods quite simple, but this simplicity limits this approach as well. One of the main issues is that PCA models generate dendric-like structures which are more typical for crystalline materials. In this regard, CCA models become a tool to overcome this difficulty and to simulate other types of materials. Specifically, CCA models are a much better choice to simulate materials which are synthesized via the sol–gel method and have a globule structure [19], such as aerogels.

**Figure 1:**
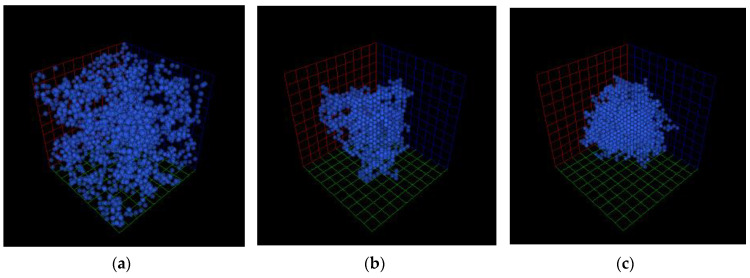
Porous structures generated by Reaction-Limited Cluster Aggregation (RLCA) (**a**), Diffusion-Limited Cluster Aggregation (DLCA) (**b**), and Ballistic Cluster–Cluster Aggregation (BCCA) (**c**) methods.

In CCA methods, all substantial particles (or globules) of a structure to be generated are placed randomly in the modelling field at the beginning. Then particles start moving and aggregating. The clusters formed in this way move in the same manner [20] as individual particles. The described motion is performed until a single cluster is formed (all particles must be included). The CCA family includes methods such as Diffusion-Limited Cluster Aggregation (DLCA), Reaction-Limited Cluster Aggregation (RLCA), and Ballistic Cluster–Cluster Aggregation (BCCA). Due to different aggregation procedures and/or motion rules, structures generated using various CCA methods have significantly different densities. For example, Figure 1 demonstrates digital structures of the same dimensions and globule size, but generated using different approaches. It can be seen that RLCA generates structures with lower density than the two other methods, and BCCA gives the densest patterns among the CCA methods. This fact can be taken as a criterion for preliminary selection of cluster-aggregation-based simulation approaches. Figure 1 shows digital porous structures generated with different CCA methods.

Within the framework of the presented work, we investigated the application of the developed models to simulate structures and predict structure-dependent properties of polyamide (PA) aerogels. Polyamides (PAs) are a class of extremely strong polymers that most notably include Kevlar^TM^. That is why PA aerogels are perspective materials. Polyamides can be prepared from diacid chlorides and diamines (Equation (1)) or from triisocyanates and multifunctional carboxylic acids (Equation (2)) [21,22,23].

R–COCl+R΄–NH_2_ → R–CONHR΄+HCl (1)

R–COOH+R΄–N=C=O → R–CONHR΄+CO_2_ (2)

This work mainly considers the modeling of the structure and properties of PA aerogels and carbon (PA-C) aerogels obtained by pyrolysis of the corresponding PA aerogels [21,22], but experimental studies are reported as well, since they are required to perform model verification.

Aerogel properties directly depend on its structure. Therefore, aerogel property prediction must be started with a simulation of a 3D structure which then is utilized for property calculations. Below the following steps of our investigation are reported: synthesis of PA and PA-C aerogels (according to procedures in the literature) [21], structure simulation of the obtained samples and modeling of their mechanical properties. The modelling was performed using “in-house” codes.

## 2. Results and Discussion

To obtain model structures that correspond to the experimental samples of polyamide aerogels, computational experiments were conducted for various sets of parameters required for the DLCA model, namely, the globule size and the sample’s porosity (Table 1).

**Figure 2:**
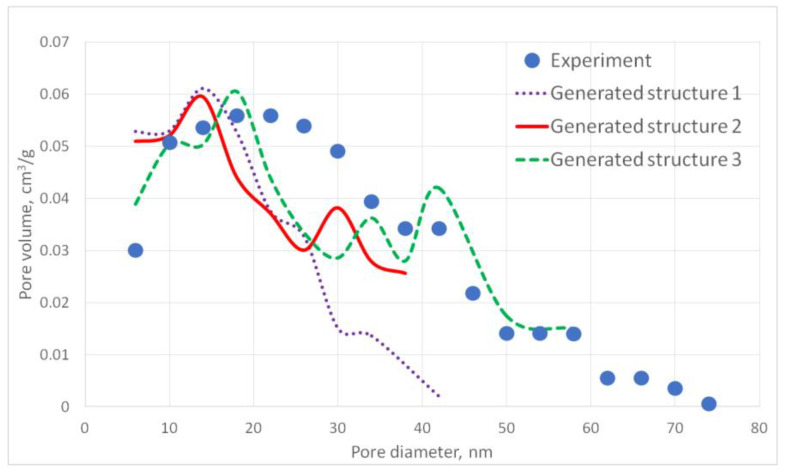
Pore size distribution curves for generated PA structures 1–3.

**Figure 3:**
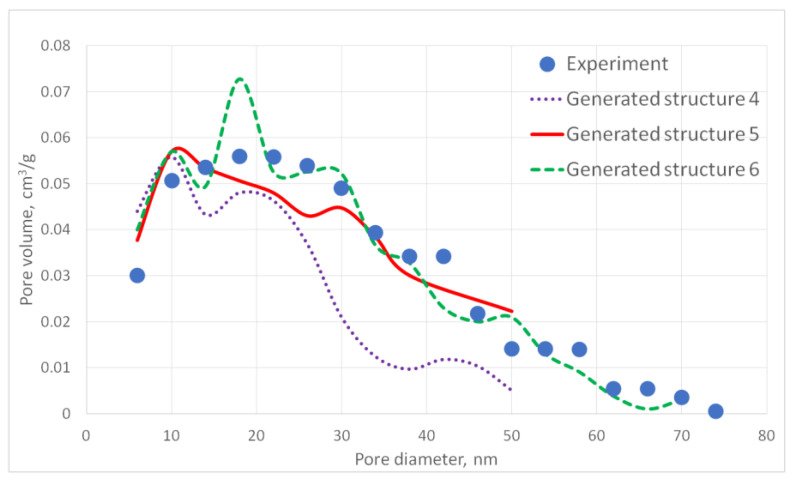
Pore size distribution curves for generated PA structures 4–6.

**Figure 4:**
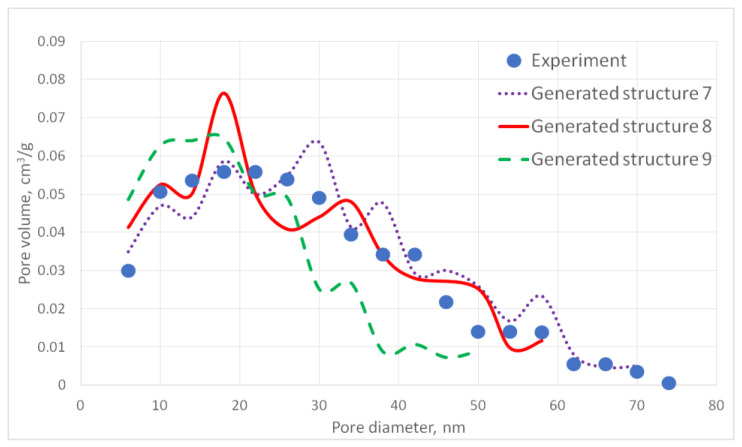
Pore size distribution curves for generated PA structures 7–9.

**Figure 5:**
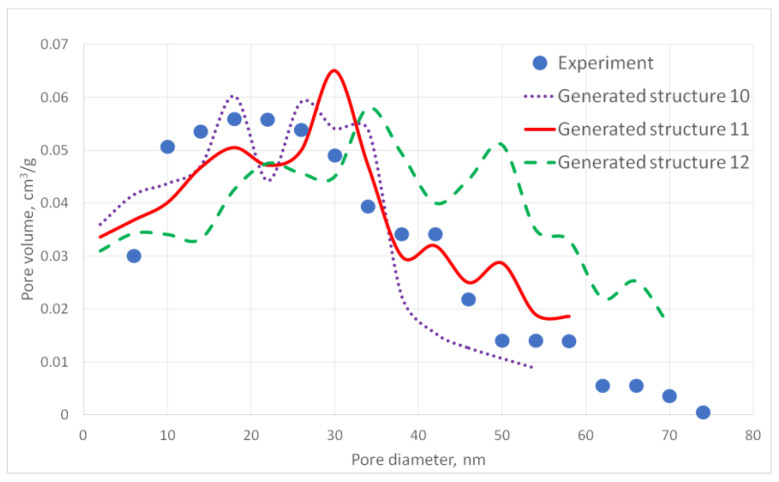
Pore size distribution curves for generated PA structures 10–12.

**Figure 6:**
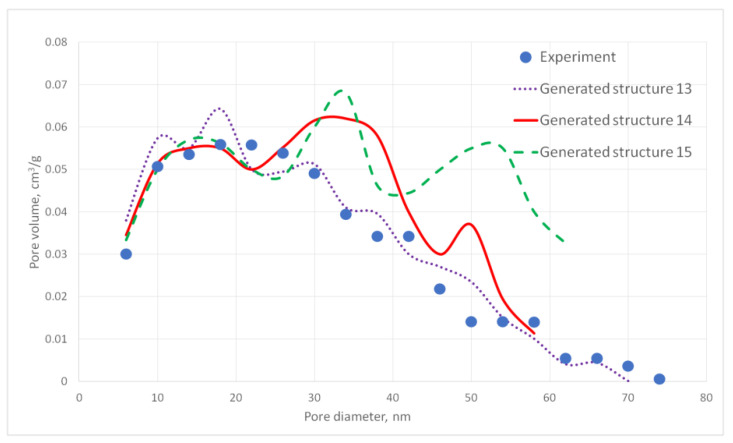
Pore size distribution curves for generated PA structures 13–15.

For the generated structures, pore size distribution and specific surface area were calculated (Figure 2, Figure 3, Figure 4, Figure 5 and Figure 6, Table 1).

The dimensions of the simulated aerogel digital structures are 400 × 400 × 400 nm. The structures are generated in 3D space, but for better illustration of changes occurring in a structure during carbonization, 2D cross-sections are provided below.

The aerogel’s pore size directly depends on the globule diameter and the sample’s porosity: as the globule diameter increases, the peaks of the pore size distribution curve shift towards larger diameters, and so does the porosity. Moreover, there are some oscillations in the calculated pore size distributions curves. This can be explained by the random character of globules motion, which generates pores with larger and less diameters. The specific surface area increases with increasing porosity and decreases with increasing diameter of the globules. The pore size distribution curve of the generated structure 13 fits the experimental data obtained for the PA sample best of all. A good agreement of the calculated (structure 13) and experimental specific surface area is reached —153 and 140 m^2^/g, respectively. The deviation of the calculated values from the experimental ones does not exceed 15%.

**Figure 7:**
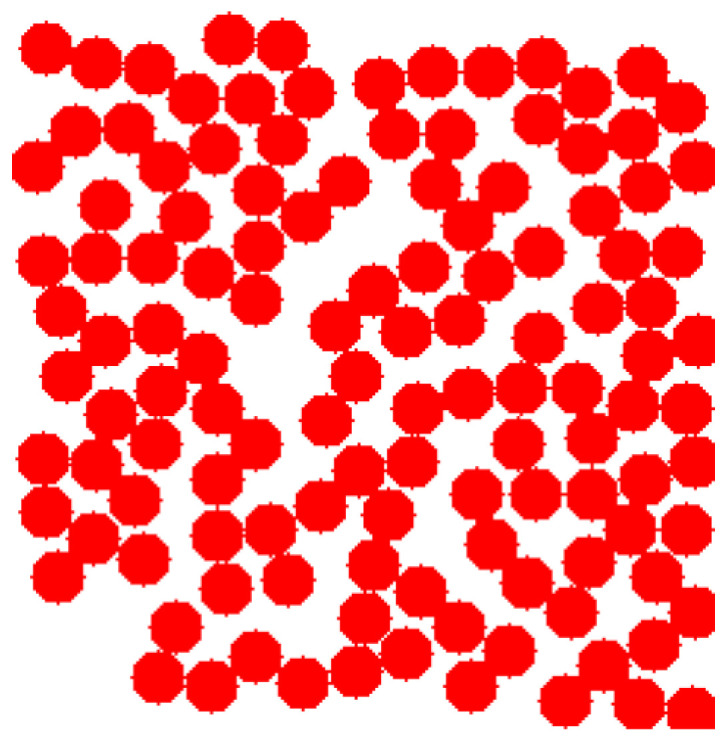
Cross-section of generated structure 13.

The cross-section of the generated structure 13 is shown in Figure 7.

**Figure 8:**
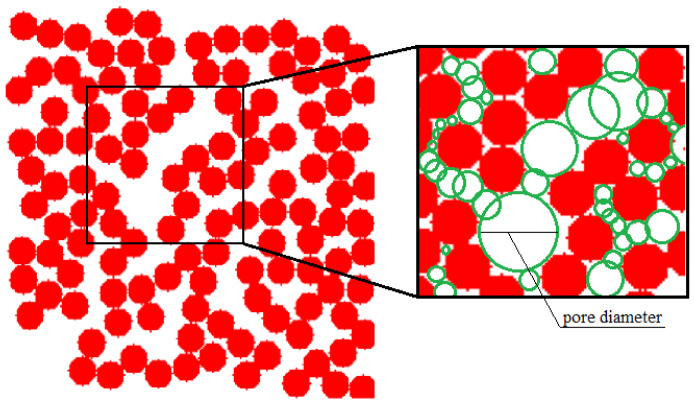
Cross-section of generated structure 13 with placed pores (green circles).

In the Figure 7 “cross-section” means that a layer of the structure with a thickness of about 1 globule was selected so that this layer forms a unified structure. The figure shows a top view of the resulting plot. The studied structures have a low porosity, so globules form (rather than a dense net) and lie on one layer, not deviating very much from the others. The generated structure 13 was used further for modeling of a carbon structure after pyrolysis and for predicting mechanical properties. Figure 8 shows part of the digital structure 13 with placed pores.

**Figure 9:**
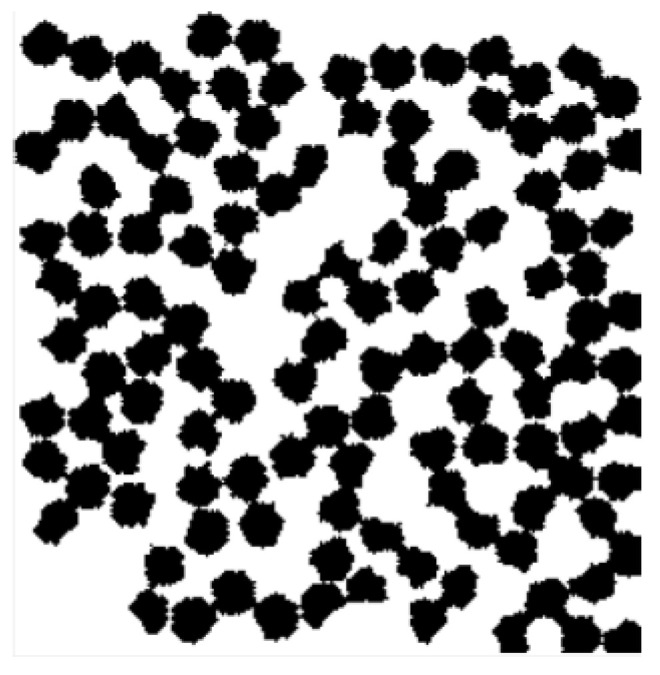
Cross-section of the generated carbon aerogel structure obtained from the generated structure 13.

Figure 8 shows how pores are placed on the generated structures (green circles). These pores increase its diameter during carbon aerogel generating. Cells with substance inside the new diameter are removed. Figure 9 shows the cross-section of the corresponding generated carbon aerogel structure.

**Figure 10:**
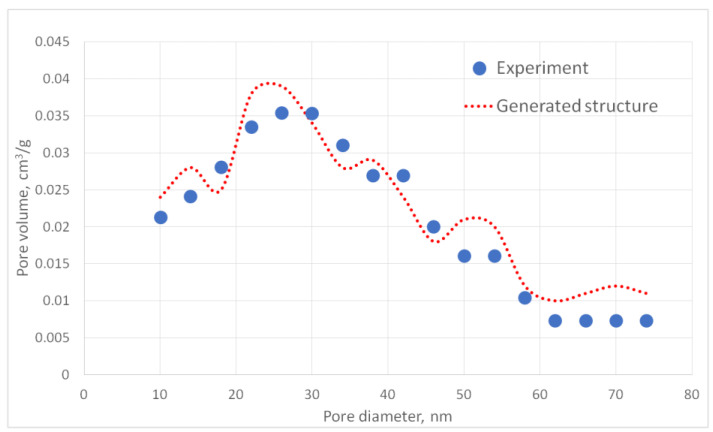
Pore size distribution curves for the experimental sample of carbon aerogel PA-C and the corresponding generated structure.

Figure 10 presents a comparison of the calculated and experimentally obtained pore size distribution curves for the carbon aerogel sample (PA-C) obtained on the basis of the PA sample.

The specific surface area of the model structure is 378 m^2^/g, which corresponds to the specific surface area of the experimental sample—341 m^2^/g.

The deviation of the model and experimental pore size distribution curves as well as the specific surface area values do not exceed 15%, which indicates that the model structure of carbon aerogel is similar to the experimental one.

Suggested structure generation models allow for the obtention of digital copies of experimental samples whose structure properties (PSD and specific surface area) match each other. They do not simulate the structure formation process in detail; their function is to reproduce the final structure based on the data that determine this structure, such as porosity and globule diameter. The obtained digital structures can be used further to calculate its properties. For the obtained generated structure 13, the value of Young’s modulus was calculated. The calculation was performed using the developed cellular automaton model. Only the load applied to the top of the sample along the OZ axis (uniaxial compression) is considered.

**Figure 11:**
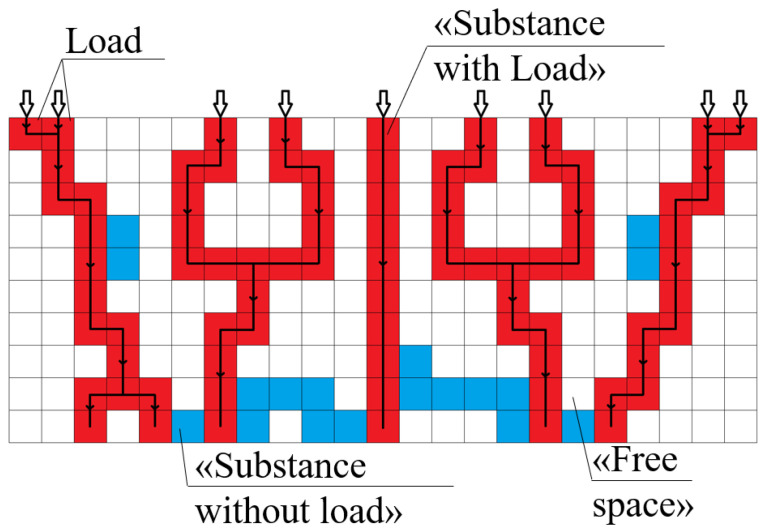
Young’s modulus calculation (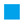
—“substance without load”, 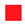
—“substance with load”, 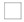
—“free space”, 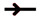
—the shortest paths).

The developed model has the following assumptions:

- The model structure of the sample for which the mechanical properties are calculated is a three-dimensional matrix. The cells of which are cubes with a unit edge length.
- Each cell can have one of three states: “substance with load”, “substance without load” or “free space”, and the state “substance with load” is characterized by material Young’s modulus (Young’s modulus of polyamide or carbon).
- Forces (imitation of the deformation process during compression) are applied to the cells on the top (first) layer of the sample and are directed vertically downwards. The suggested model does not require specification of stress distribution.
- No force is applied to cells with the “free space” state.
- Cells of the top (first) layer that do not have the “free space” state are considered as “points of force application”; cells of the bottom (last) layer that do not have the “free space” state are considered as “points of support”.
- The load created by the applied forces goes along the shortest sequence of cells between the top (first) and the bottom (last) layers (Figure 11).

The input parameters of the model are an aerogel modelled structure, thus, the number of cells and their initial states depend on the structure generated using the DLCA model or the model of carbon aerogel structure (“free space” cells of the aerogel structure correspond to the “free space” of the cellular automata model to calculate Young’s modulus, and “polyamide” or “carbon” cells of the aerogel structure corresponds to the “substance with load” or “substance without load” of the cellular automata model to calculate Young’s modulus) and Young’s modulus of the material (polyamide or carbon).

Young’s modulus of the physical body can be calculated as the ratio of the applied vertical load to the multiplication of the surface area to which the load is applied and the relative elongation of the body:

(3) $$E_{structure}=\frac{F}{\varepsilon_{structure}S_{structure}}$$

where *F* is the force applied to the upper surface of the sample and directed vertically downward, N; $\varepsilon_{structure}$ is the relative strain of the structure; $S_{structure}$ is the area of the upper surface of the structure, m^2^; *F* is a predetermined value; and $S_{structure}$ is a known structure parameter.

Thus, the only unknown value is the strain, which depends on the structural characteristics and composition of the sample. Since the studied samples have a complex heterogeneous structure, they are divided into elementary layers. Index *j* corresponds the structure layer, and index *i* corresponds to the cell on layer *j*. The total strain is calculated as the average strain of all layers:

(4) $$\varepsilon_{structure}=\frac{\sum_{j=1}^{N_{z}}\varepsilon_{j}}{N_{z}}$$

where $\varepsilon_{j}$—relative strain of the layer *j*; *N_z_*—the total number of layers in digital structure.

Each layer also consists of a solid and pores filled with air. Therefore, each layer is divided into elementary sections (cells), each of which contains either a solid substance and air (“substance with load” or “substance without load” and “free space”). Then, the relative stress of the layer can be calculated as the average strain of each cell with a solid substance of this layer which depends on the load (the cells with or without load). For each layer, the relative strain is calculated separately:

(5) $$\varepsilon_{j}=\frac{\sum_{i=1}^{N_{load}^{j}}\varepsilon_{i}}{N_{load}^{j}},$$

where $\varepsilon_{i}$—relative strain of the cell *i*; $N_{load}^{j}$—the total number of layer *j* cells that have “substance with load”.

Not every solid cell is affected by the load—some of them are those branches of the frame that do not support the structure. The assumption that applied forces go along the shortest sequence of cells between the top (first) and the bottom (last) layers allows for the state of “substance with load” to be set to the cells, which leads to the shortest path from each “point of force application” to the nearest “point of support”. The state of other solid cells is set as “substance without load”. These “substance without load” cells are not considered in calculations of the layer strain, which considers the presence of gaps in the structure as its porosity increases. For “substance with load” cells, $\varepsilon_{i}$ is calculated with the following formulas:

(6) $$\varepsilon_{i}=\frac{F}{E_{i}S_{load}^{j}}$$

(7) $$S_{load}^{j}=\;N_{load}^{j}\times l_{0}^{2},$$

where $E_{i}$—Young’s modulus of the cell *i* material (for example, polyamide), MPa; $S_{load}^{j}$—the layer *j* area, m^2^; $N_{load}^{j}$—the total number of layer *j* cells that have “substance with load”; and $l_{0}$—the size of the cell, m.

The mechanical properties of aerogels directly depend on their density. In [24], it was shown that for porous materials, Young’s modulus correlates to the relative density according to the power law. It is assumed that this is due to the presence of gaps in the structure of the aerogel which do not take the load. The suggested model considers only elastic deformations, finds gaps in the structure and does not consider them in Young’s modulus calculations.

In the developed model, horizontal force transmitting is not considered. The studied structures have low porosity and have a dense structure that forms connected net. Therefore, assumptions about horizontal forces are valid for these structures, since there are few uncompensated horizontal effects that can be neglected in the studied structures.

Thus, the suggested model for calculating Young’s modulus by a digital structure works for a certain range of porous materials. For materials whose structure differs from the structures studied in the current work, this model will lose accuracy and require further extension.

Young’s modulus was calculated for the generated structure corresponding to the PA sample. Young’s modulus for polyamide was 2200 MPa. This value was used in calculations of the model. The experimental value of Young’s modulus is E_exp_ = 9 ± 4 MPa, and the calculated value is E_calc_ = 8 MPa. It can be concluded that Young’s modulus of the model and experimental samples corresponds within the limits of measurement accuracy. Thus, the developed models can be used to obtain model structures of polyamide and carbon aerogels derived from them and to predict their properties, such as Young’s modulus.

## 3. Conclusions

In this work, polyamide (PA) aerogel samples were produced via reaction of 4,4’,4”-tris(isocyanatophenyl)methane with pyromellitic acid. PA aerogels were pyrolyzed to obtain carbon (PA-C) aerogels. Both PA and PA-C samples were analyzed with nitrogen porosimetry. Nitrogen porosimetry data were used to obtain structure characteristics of the samples, to plot pore size distribution with the BJH method, and to obtain specific surface area with BET. Skeletal and bulk density using He pycnometry were also obtained. For PA samples, their mechanical properties (Young’s modulus) were investigated.

Two cellular automata were developed to generate virtual structures of the studied aerogels—cellular automata based on a modified DLCA method to generate a virtual structure PA and the original cellular automata model which reproduces the pyrolysis process inside porous materials consisting of organic substances.

For PA and PA-C virtual structures, Young’s modulus was calculated. The calculated Young’s modulus of PA virtual structure corresponds to that of the experiment.

The developed models allow one to generate structures which correspond to experimental samples. As discussed above, these structures can be applied as input parameters for the models which predict different properties of the aerogel, such as Young’s modulus. The developed model can be applied to the modeling of other PA aerogels to a certain extent. If the concentration of chemicals that are used to synthesize the aerogel structure is significantly reduced, this can lead to structure type changes. Correspondingly, applicability of the DLCA approach in this case is arguable. However, to the best of our knowledge, such low-density PA aerogels are out of practical use. Thus, the considered cellular automata models allow one to reduce the amount of experimental research to obtain aerogels with certain properties that reduce the required resource and time costs. Moreover, the suggested models can be used in unison with other models for different scales, for example, molecular dynamics and the finite element method. This will allow for the utilization of multiscale modelling of the structure from one particle to the whole sample scale.

## 4. Materials and Methods

Synthesis of PA aerogels was performed using Desmodur RE triisocyanate kindly provided by Covestro Deutschland GA (Leverkusen, Germany). It was supplied as a 27% *w*/*w* solution of 4,4’,4”-tris(isocyanatophenyl)methane (TIPM) in ethyl acetate and was used as received. Tetrahydrofuran (THF) was purchased from Thermo Fisher Scientific (Waltham, MA, USA), and it was distilled from Na/benzophenone prior to use. 1,2,4,5-benzenetetracarboxylic acid (pyromellitic acid, PMA, 96%) was purchased from Sigma-Aldrich (Sigma-Aldrich Chemie GmbH, Steinheim, Germany), and it was dried at 120 °C overnight.

### 4.1. Synthesis of Polyamide (PA) Aerogels From TIPM and PMA In THF (Sample PA)

**Figure 12:**
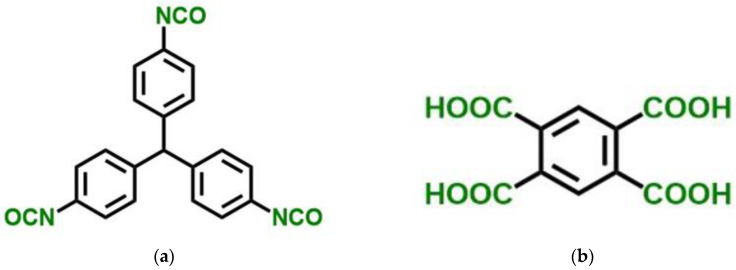
Structures of tris(4-isocyanatophenyl)methane (TIPM; Desmodur RE) (**a**) and pyromellitic acid (PMA) (**b**). Groups shown in green react according to Equation (2).

PA aerogel monoliths were synthesized from TIPM (Desmodur RE, Figure 12) and pyromellitic acid (PMA, Figure 12), according to Equation (2). Samples were prepared according to a procedure described in [21]. In brief, PMA (3.05 g, 12.0 mmol) was placed in round bottom flask and was dissolved in anhydrous THF (40 mL for 20% *w*/*w* of total monomer concentration). Desmodur RE (TIPM, 21.3 mL, 21.8 g, 16.0 mmol) was added. The resulting sol was stirred mechanically (400 rpm) at room temperature under Ar for 15 min, and it was poured in polypropylene molds 1 cm in diameter. Molds were kept at room temperature for 24 h for gelation and aging. Gels were removed from the molds directly into fresh acetone, were washed with fresh acetone (5 times using 4 times the volume of each gel for each wash), and were dried with liquid CO_2_ in an autoclave taken out at the end as a supercritical fluid (SCF).

It is noted in passing that, because of the mechanism of formation of these materials, in reality, they are random copolymers with polyamide as the main component and the corresponding polyurea and polyimide as minor components [21].

Materials were characterized in terms of their skeletal densities using He pycnometry, bulk densities, BET surface areas and BJH pore size distribution using N_2_ sorption porosimetry, and morphology using SEM.

### 4.2. Conversion of PA Aerogels to Carbon Aerogels (PA-C)

Carbon (PA-C) aerogels were prepared from the pyrolysis of PA aerogels. Samples were prepared according to a procedure described in [21]. In brief, the PA aerogels prepared as described in Section 4.1 above were processed using an MTI GSL-1800X-KS60 tube furnace (Al_2_O_3_ ceramic >99.9% pure, 54/60 mm inner/outer diameters, 300 mm heating zone). The temperature was raised to 800 °C at 2.5 ^o^C min^−1^ under flowing Ar (150 mL min^−1^) for 5 h.

PA-C aerogels were characterized in terms of their skeletal densities using He pycnometry, bulk densities, BET surface areas and BJH pore size distribution using N_2_ sorption porosimetry, and morphology using SEM.

### 4.3. Aerogel Structure Modeling

#### 4.3.1. Polyamide (PA) Aerogel Structure Modeling

**Figure 13:**
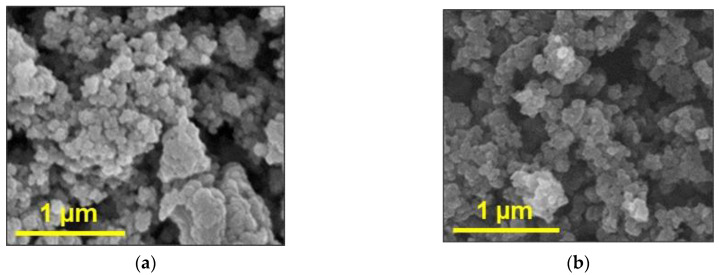
SEM images of polyamides (PA) (**a**) and PA aerogels and carbon (PA-C) (**b**) samples.

**Figure 14:**
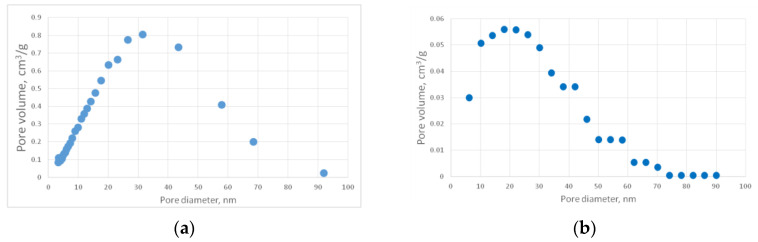
Differential (**a**) and recalculated (**b**) pore size distribution curves of the PA sample.

For this work, PA aerogels were synthesized from TIPM (Desmodur RE, Figure 12) and pyromellitic acid (PMA, Figure 12), as described in Section 4.1 above. The obtained structural characteristics and mechanical properties of produced PA aerogels, such as skeletal and bulk densities, pore size distribution, specific surface area, and Young’s modulus, were utilized to create corresponding structure models, as well as to conduct their validation. Figure 13a shows a representative SEM image of the PA sample. Pore size distribution curves, obtained with nitrogen porosimetry, are shown in Figure 14. Measured characteristics of the PA sample are provided in Table 2. 

**Figure 15:**
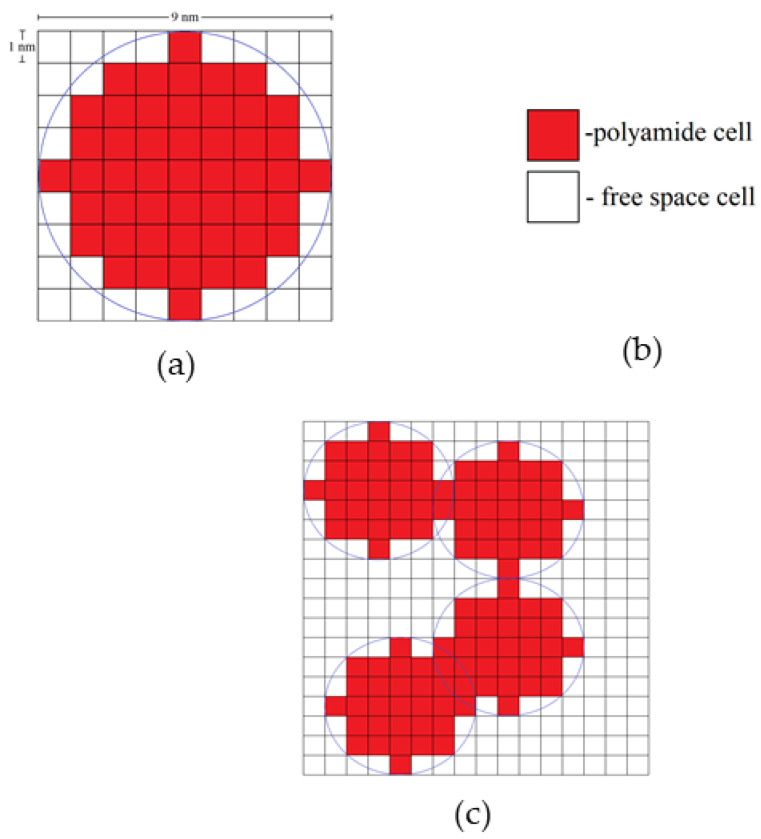
Two-dimensional globule with a diameter of 9 nm (**a**); types of cells in developed the DLCA model (**b**) and aggregated 2D globules with a diameter of 7 nm (**c**).

Figure 14a shows the differential curve of the PA sample pore size distribution. This curve was transformed from “dV/dlog(D)-pore diameter” dimension to “pore volume - pore diameter” (Figure 15b) because it has better visibility and physical interpretation. The transformed plot was obtained with the following steps:

Initially, we had a set of pair values $dV/dlog{\left(D\right)}_{n}$, where *n* is the number of experimental points; *D* is the pore diameter, nm; and *V* is the volume of pores, cm^3^. From these sets, we can obtain a set of pair values $dV_{n}-D_{n}$ with the following formula:

(8) $$dV_{n}=\frac{dV_{n}}{dlog{\left(D\right)}_{n}}\times \left(\log{\left(D\right)}_{n}-\log{\left(D\right)}_{n-1}\right)$$

After that, we can obtain a cumulative curve *V_n_–D_n_* by summarizing the volume at each point:

(9) $$V_{n}=dV_{n}+V_{n-1}$$

When calculating the pore size distribution of virtual structures, it is possible to calculate pore values only at equal intervals of diameter since the virtual structure is digital. Therefore, for the experimental pore size distribution curve, it is necessary to interpolate and find the curve values for those diameter values that correspond to the values of the pore size distribution curve of the digital structure. In this work, the initial pore diameter for virtual structures’ pore size distribution is 6 nm with a further step of 4 nm. Volume values for these pore diameters were calculated with the assumption that the curve between two experimental points is linear. Thus, we can find the volume value *dV_m_* for the diameter *D_m_* where *D*_*n*−1_ < *D_m_* < *D_n_* using the linear equation, where *n* is the number of experimental points of the pore size distribution curve, and *m* is the number of interpolated points:

(10) $$dV_{m}=\frac{V_{n}-V_{n-1}}{D_{n}-D_{n-1}}D_{m}+V_{n-1}-\frac{V_{n}-V_{n-1}}{D_{n}-D_{n-1}}D_{n-1}$$

Using this procedure, the recalculated (Figure 14b) pore size distribution curve was obtained.

It can be seen in the SEM image (Figure 13a) that the structure of the experimental sample consists of a distinct globules, the size of which varies in the range from 25 to 40 nm. The pore size distribution curve (Figure 14b) demonstrates that most of the pores have diameters of about 20 nm. The sample porosity is 80% *v*/*v*. In addition, Figure 13a shows pores whose diameters are larger than 300 nm. It is worth mentioning that these pores cannot be measured using the nitrogen porosimetry method.

To obtain model structures of polyamide aerogels, a cellular automaton model of diffusion-limited cluster aggregation (DLCA) was used. The selection of the model is based on the analysis of SEM images of PA aerogels. As mentioned above, different CCA models generate structures with different densities, and the DLCA method is that which allows generating medium dense patterns. Since the investigated PA aerogels have a moderate density of structure, the DLCA model was chosen.

The model has the following assumptions:

- The modeling space consists of equal size cells;
- Each cell can have one of two states: “polyamide” or “free space”;
- At the beginning of the simulation, only individual globules (particles) are present in the simulated space, which then aggregate into a single structure;
- Each globule (particle) is a collection of neighboring cells with the “polyamide” state and has a round shape;
- Globules (particles) move chaotically, imitating the Brownian movement, and they are not influenced by any external forces.

Input parameters of the model are globules’ diameter and porosity of the structure. Globules are placed on the simulation field and do not overlap each other. The initial velocity vector for each globule is randomly specified. The velocities have the same absolute value equal to one cell per one iteration on a cubic lattice. Globules begin to move and aggregate when they collide. The cycle is stopped when all particles aggregate in a single cluster.

The amount of polyamide cells is calculated from the porosity of the experimental sample:

(11) $$N_{pa}=\left(1-\prod \right)N_{field}$$

where $N_{field}$ is the total number of cells and ∏ is the porosity. The total number of cells is the variable parameter of the model. For example, if the size of three-dimensional field is 400 cells, the total number of cells is 400^3^ = 64,000,000.

Each globule consists of a certain number of polyamide cells. For example, a two-dimensional (2D) globule with diameter 9 nm consists of 49 cells. A three-dimensional (3D) globule contains 257 polyamide cells. Figure 15 shows the types of cells in the developed DLCA model, a single globule, and an aggregate of globules.

The result of the simulation is a single aerogel structure consisting of globules of a given diameter.

The structural characteristics of the sample—the pore size distribution and specific surface area—were obtained using nitrogen porosimetry, which does not allow for the consideration of pores with a diameter greater than 300 nm. Thus, those parts of the structure were modeled to contain only pores with a diameter of less than 300 nm, since such model structures can be compared with an experimental sample by a specific surface area and pore size distribution.

The porosity of such areas does not equal the total porosity of the sample and is calculated by:

(12) $$V_{sol}=\left(1-\prod \right)V_{total}$$

(13) $$V_{total}=m/\rho_{bulk}$$

where $V_{sol}$ is the volume of the sample’s solid part, ∏ is the sample porosity, $V_{total}$ is the sample volume, *m* is the sample mass, and $\rho_{bulk}$ is the bulk density of the sample. The pore volume obtained from nitrogen porosimetry data is equal to $V_{por}$ = $V_{nit}\cdot m$, where $V_{nit}$ = 0.53 cm^3^/g. Consequently, the porosity of sample parts that do not contain pores greater than 300 nm can be calculated using the following correlation:

(14) $$\prod_{nit}=\frac{V_{por}}{V_{sol}+V_{por}}=\;\frac{V_{nit}}{\left(1-\prod \right)/\rho_{bulk}+V_{nit}}$$

where $\prod_{nit}$ is the porosity of sample parts that do not contain pores greater than 300 nm, and $V_{nit}$ is the pore volume obtained from nitrogen porosimetry data referred to the sample mass. The calculated ∏*_nit_* value for the PA sample is 44%. This porosity was used further as an input parameter of the CA model for PA aerogel structure generation.

#### 4.3.2. Carbon Aerogel Structure Modeling

**Figure 16:**
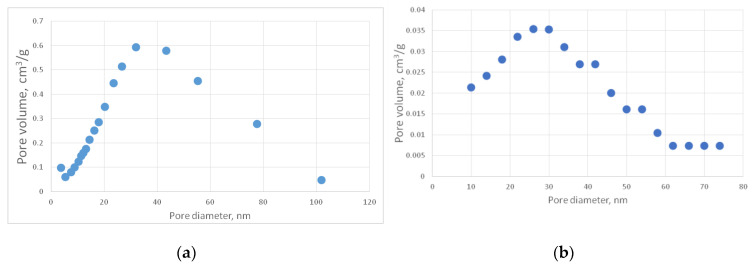
Differential (**a**) and recalculated (**b**) pore size distribution curves of the PA-C sample.

PA aerogels were carbonized using the tube furnace as described in Section 4.2. For the obtained PA-C samples, the pore size distribution and specific surface area were measured. Figure 13b shows a representative SEM image of the PA-C sample. Figure 16 shows the corresponding pore size distribution curves. Table 3 contains the measured characteristics of the PA-C sample.

For better visibility, a PA-C differential curve was recalculated using the same procedure as for the PA sample (Figure 16b).

To generate a model structure of carbon aerogels, a cellular automaton model was developed which reproduces the pyrolysis process inside porous materials consisting of organic substances.

At the first step of the simulation algorithm, identification of all pores existing in the generated structure is carried out. At subsequent steps, each pore can increase its diameter with a certain probability.

The developed model has the following assumptions:

- The modeling space consists of equal sized cells;
- Each cell can have one of three states: “polyamide”, “carbon”, or “free space”;
- Each pore is a set of neighboring cells having the state of “free space”, has a spherical shape, and may overlap with other pores;
- Pyrolysis of organic substance occurs along the boundary between “polyamide” and “free space”;
- During pyrolysis, there is an increase in the pores and the removal of “polyamide” inside the new pore area.
- Each pore increases with a variable probability, which depends on the pore diameter as follows:

*P* = 0.02∙*d_pore_*/*d*_100_ (15)

where *P* is the pore diameter’s probability of increase, *P* ∈ [0,1]; *d_pore_* is the pore diameter, *d*_100_ = 100 nm. This relationship was obtained empirically by carrying out a large number of computational experiments with test structures. Silica–carbon composites obtained from silica–resorcinol formaldehyde aerogels via pyrolysis were used as test structures. Pyrolysis was carried out inside an electric furnace under 700 °C.

The algorithm of the developed model can be represented by the following steps:

1. Identification of all pores.
2. The diameter of each pore is increased with the calculated probability by the fixed step, and all the “polyamide” inside pores with new diameters is removed.
3. Step 2 is repeated until the required fraction of “polyamide” is removed.
4. Changing the state all “polyamide” cells to “carbon”.

**Figure 17:**
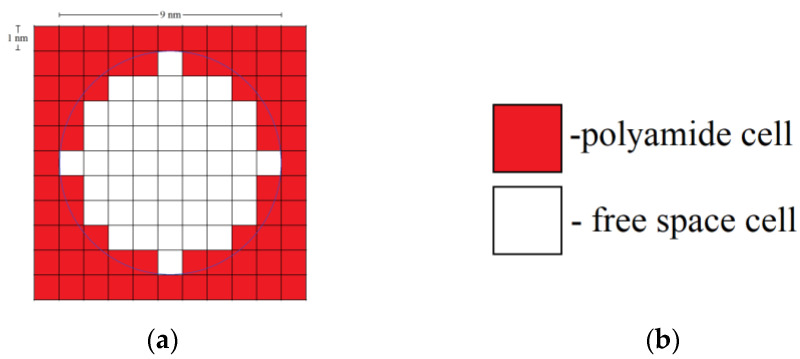
Two-dimensional pore with a diameter of 9 nm (**a**); types of cells in the developed model of the carbon aerogel structure (**b**).

In the pyrolysis model program, pores are recognized as follows: In each empty cell, the program tries to place a pore with a certain diameter where the considered cell acts as the center of the spherical pore. If there are cells with substance inside the pore or the placed pore overlaps with the others too much, then the pore is removed. Otherwise, this pore is not removed from the structure, and its cells are marked as “pore cells”. After all the structure empty cells are considered, a certain diameter is reduced and the procedure is repeated. Algorithms are carried out until all possible pore diameters from the maximum level to the minimum one are considered. The pore volume is calculated from the number of pore cells that belong to each diameter. Figure 17 shows the cell types of the developed model and a pore.

The input parameters of the model are an aerogel modelled structure (thus, the number of cells and its initial states corresponds the structure generated with the DLCA model) and the amount of substance that must be removed. The amount of substance that must be removed is calculated from the amount of carbon in the experimental PA-C sample.

## Figures and Tables

**Table 1:** DLCA model parameters for polyamide aerogel structure generation and specific surface area of generated structures.

Generated Structure ID	Globule Diameter, nm	Porosity, %	Calculated Specific Surface Area, m^2^/g	Experimental Specific Surface Area, m^2^/g
1	22	45	201	140
2	22	50	220	
3	22	55	245	
4	26	45	189	
5	26	50	197	
6	26	55	223	
7	30	45	176	
8	30	50	192	
9	30	55	219	
10	34	45	163	
11	34	50	181	
12	34	55	222	
13	38	45	153	
14	38	50	176	
15	38	55	190	

**Table 2:** Measured characteristics of the PA sample.

Sample	Bulk Density, g/cm^3^	Skeletal Density, g/cm^3^	Specific Surface Area (Micropore Surf. Area), m^2^/g	Pore Volume, cm^3^/g	Young’s Modulus, MPa
PA	0.272 ± 0.007	1.342 ± 0.003	140 (20)	0.54	9 ± 4

**Table 3:** Measured characteristics of the PA-C sample.

Sample	Bulk Density, g/cm^3^	Skeletal Density, g/cm^3^	Specific Surface Area (Micropore Surf. Area), m^2^/g	Pore Volume, cm^3^/g	Young’s Modulus, MPa
PA-C	0.416 ± 0.005	1.983 ± 0.009	341 (218)	0.43	N/A
